# Supplementary material for: Noise-induced barren plateaus in variational quantum algorithms
Source: Nat Commun. 2021 Nov 29;12:6961. doi: 10.1038/s41467-021-27045-6 (PMC8630047; doi:10.1038/s41467-021-27045-6)
Supplement: Supplementary file 1 — Supplementary Information [file 41467_2021_27045_MOESM1_ESM.pdf]

Supplementary Information for *Noise-induced barren plateaus in variational quantum algorithms*

In this Supplementary Information we provide proofs for the main results of the manuscript "Noise-induced barren plateaus in variational quantum algorithms". In Supplementary Note 1 we first present some definitions and lemmas which will be useful in deriving our results. We point readers to [1, 2] for additional background. Then, in Supplementary Note 2 we present a detailed proof of our main result Theorem 1. Supplementary Notes 3 and 4 respectively contain the proofs for Lemma 1 on cost concentration, and Remark 1 on a generalization to correlated (degenerate) parameters. We present our proof for Remark 2 on extensions to the noise model to k-local noise in Supplementary Note 5 and our proofs of Corollaries 2 and 3 on application-specific results in Supplementary Note 6. In Supplementary Note 7 we discuss our Remark 3 on a construction where the cost function is summed over some dataset. Finally, the proof for Proposition 1 on measurement noise is detailed in Supplementary Note 8.

Supplementary Note 1 - Preliminaries

A. Definitions

Quantum states. Given some choice of Hilbert space $\mathcal{H}$, we denote the set of density operators as $\mathcal{S}(\mathcal{H})$.

Pauli expansion. We note that one can always expand H_{lm} and O in the Pauli basis as

$$H_{lm} = \sum_i \eta^i_{lm}\sigma^i_n = c^0_{lm}\sigma^0_n + \boldsymbol{\eta}_{lm}\cdot\boldsymbol{\sigma}_n\,, \tag{1}$$

$$O = \sum_i \omega^i\sigma^i_n = \omega^0\sigma^0_n + \boldsymbol{\omega}\cdot\boldsymbol{\sigma}_n\,. \tag{2}$$

where now $\sigma^i_n \in \{\mathbb{1}, X, Y, Z\}^{\otimes n}\backslash\{\mathbb{1}^{\otimes n}\}$ length-n Pauli strings. Here we remark that for the sake of simplicity we have made a subtle change in notation as now $\sigma^0_n = \mathbb{1}^{\otimes n}$ is treated on a separate footing. With this notation, $\boldsymbol{\sigma}_n, \boldsymbol{\eta}_{lm}, \boldsymbol{\omega}$ are real vectors of length $2^{2n}-1$ and run over indices $i \in [4^n-1]$. Moreover, we recall that we have defined $N_{lm} = |\boldsymbol{\eta}_{lm}|$, and $N_O = |\boldsymbol{\omega}|$ as the number of non-zero elements in each respective vector. Furthermore, note that we can always set $\omega^0 = 0$ and $\eta^0_{lm} = 0$ for all lm. This does not lose us generality in our setting as a non-zero ω^0 corresponds to a trivial measurement, while a non-zero η^0_{lm} simply leads to a different choice in the Hamiltonian normalization.

Vector norms. In what follows we use the usual definitions of the p-norms such that $\|\boldsymbol{a}\|_\infty \equiv \max_i |a_i|$ is the largest element of vector $\boldsymbol{a}$ and $\|\boldsymbol{a}\|_2 \equiv \sqrt{\sum_i |a_i|^2}$ is the Euclidean norm.

Setting for our analysis. As shown in Supplementary Figure 1 we break down the circuit into L unitaries preceded and followed by noisy channels acting on all qubits. Let ρ_0 and ρ_l respectively denote the input state and the state obtained after the l-th unitary. Let $\mathcal{N} = \mathcal{N}_1 \otimes \cdots \otimes \mathcal{N}_n$ denote the n-qubit noise channel. Then the noisy cost function $\widetilde{C}$, defined as the expectation value of an operator O, can be represented as follows:

$$\widetilde{C} = \mathrm{Tr}\Big[O\,\big(\mathcal{N}\circ\mathcal{U}_L(\boldsymbol{\theta}_L)\circ\mathcal{N}\circ\cdots\circ\mathcal{U}_2(\boldsymbol{\theta}_2)\circ\mathcal{N}\circ\mathcal{U}_1(\boldsymbol{\theta}_1)\circ\mathcal{N}\big)(\rho_0)\Big]\,, \tag{3}$$

where the l-th unitary channel $\mathcal{U}_l(\boldsymbol{\theta}_l)$ implements the unitary operator

$$U_l(\boldsymbol{\theta}_l) = \prod_m e^{-i\theta_{lm}H_{lm}}W_{lm}\,. \tag{4}$$

Here we recall that $\boldsymbol{\theta}_l = \{\theta_{lm}\}$ are continuous parameters and W_{lm} denote unparameterized gates.

Noise model. We consider a noise model where local Pauli noise channels $\mathcal{N}_j$ act on each qubit j before and after each unitary $U_l(\boldsymbol{\theta}_l)$. The action of $\mathcal{N}_j$ on a local Pauli operator $\sigma \in \{X, Y, Z\}$ can be expressed as

$$\mathcal{N}_j(\sigma) = q_\sigma\sigma\,, \tag{5}$$

where $-1 < q_X, q_Y, q_Z < 1$. Here, we characterize the noise strength with a single parameter

$$q = \sqrt{\max\{|q_X|, |q_Y|, |q_Z|\}}\,. \tag{6}$$

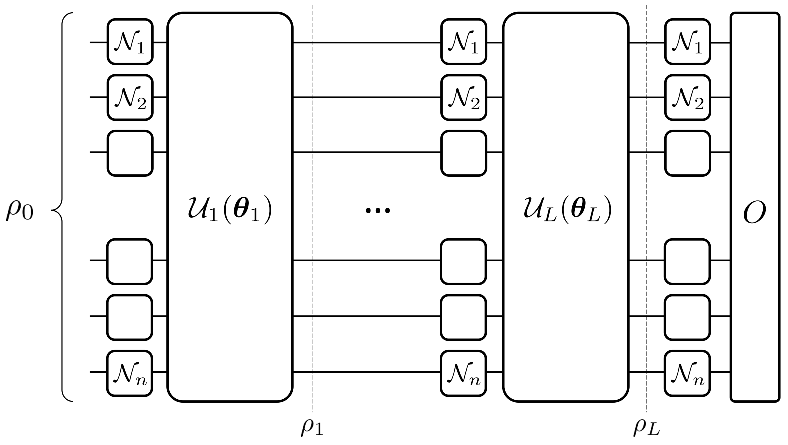

Supplementary Figure 1. **Setting for our analysis.** An n-qubit input state ρ_0 is sent through a variational ansatz $U(\boldsymbol{\theta})$ composed of L unitary layers $U_l(\boldsymbol{\theta}_l)$ sequentially acting according to Eq. (4). Here, $\mathcal{U}_l$ denotes the quantum channel that implements the unitary $U_l(\boldsymbol{\theta}_l)$. The parameters in the ansatz $\boldsymbol{\theta} = \{\boldsymbol{\theta}_l\}_{l=1}^L$ are trained to minimize a cost function that is expressed as the expectation value of an operator O as in Eq. (3). We consider a noise model where local Pauli noise channels $\mathcal{N}_j$ act on each qubit j before and after each unitary. We denote the state obtained after l applications of noise followed by unitary as ρ_l.

Representation of the quantum state. Here we will use the Pauli representation of an n qubit state

$$\rho = \frac{1}{2^n}\left(\mathbb{1}^{\otimes n} + \boldsymbol{a}\cdot\boldsymbol{\sigma}_n\right), \tag{7}$$

where $a_i = \langle \sigma_n^i \rangle = \text{Tr}[\rho\, \sigma_n^i]$. The state ρ can then be represented by a vector $\boldsymbol{a}$ of length $(4^n - 1)$, with elements a_i, which we will refer to as the Pauli coefficients.

Recalling that ρ_l is the state obtained after the application of the l-th unitary, we employ the notation $a_i^{(l)}$ for its Pauli coefficients. That is, we explicit write the output of layer l as

$$\rho_l = \frac{1}{2^n}(\mathbb{1}^{\otimes n} + \sum_{i=1}^{2^{2n}-1} a_i^{(l)}\, \sigma_n^i) = \frac{1}{2^n}(\mathbb{1}^{\otimes n} + \boldsymbol{a}^{(l)}\cdot \boldsymbol{\sigma}_n). \tag{8}$$

B. Useful lemmas

Here we present some supplementary lemmas which will be useful in deriving our main results.

Supplementary Lemma 1. *(Pauli coefficients under unitary transformations) Let Λ be an n-qubit operator whose Pauli basis decomposition is*

$$\Lambda = \lambda_0 \mathbb{1}^{\otimes n} + \boldsymbol{\lambda}\cdot\boldsymbol{\sigma}_n, \tag{9}$$

where $\lambda_0 \in \mathbb{R}$ and $\boldsymbol{\lambda} \in \mathbb{R}^{2^{2n}-1}$. Then $\|\boldsymbol{\lambda}\cdot\boldsymbol{\sigma}\|_p$ is invariant under the unitary transformation $\Lambda \to U\Lambda U^\dagger$ for any unitary operator U. In particular, this also implies $\|\boldsymbol{\lambda}\|_2$ is invariant under unitary transformations.

Proof. Denote the new Pauli coefficients after unitary transformation as $\boldsymbol{\lambda}'$. We note the first term in Eq. (9) is invariant under such transformations. Thus

$$\boldsymbol{\lambda}'\cdot\boldsymbol{\sigma} = U(\boldsymbol{\lambda}\cdot\boldsymbol{\sigma})U^\dagger \tag{10}$$

and so unitary invariance of $\|\boldsymbol{\lambda}\cdot\boldsymbol{\sigma}\|_p$ follows from the unitary invariance of Schatten norms. To see the unitary invariance of $\|\boldsymbol{\lambda}\|_2$ note that for $p = 2$ we have

$$\|\boldsymbol{\lambda}\cdot\boldsymbol{\sigma}\|_2 = \sqrt{\text{Tr}\left[(\boldsymbol{\lambda}\cdot\boldsymbol{\sigma})^2\right]} \tag{11}$$

$$= \sqrt{\text{Tr}\left[\boldsymbol{\lambda}\cdot\boldsymbol{\lambda}\, \mathbb{1}^{\otimes n}\right]} \tag{12}$$

$$= 2^{n/2}\|\boldsymbol{\lambda}\|_2 \tag{13}$$

and thus unitary invariance of $\|\boldsymbol{\lambda}\cdot\boldsymbol{\sigma}\|_2$ implies unitary invariance of $\|\boldsymbol{\lambda}\|_2$. □

Supplementary Lemma 2. *(Pauli coefficients under noise) Consider an operator* Λ *of the form* (9). *Under the action of a noise channel* $\mathcal{N}$ *of the form in Eq.* (5) *on a single Pauli string* σ_n^i *we have*

$$|\lambda_i'| \leqslant q^2 |\lambda_i| \,, \tag{14}$$

where we define λ_i' *as the coefficient such that* $\lambda_i' \sigma_n^i = \mathcal{N}(\lambda_i \sigma_n^i)$. *Additionally, we also have*

$$\|\boldsymbol{\lambda}'\|_p \leqslant q^2 \, \|\boldsymbol{\lambda}\|_p \,. \tag{15}$$

for all $1 \leqslant p \leqslant \infty$.

Proof. The effect of a single layer of noise can be expressed as follows

$$\lambda_i \xrightarrow{\mathcal{N}} q_X^{x(i)} q_Y^{y(i)} q_Z^{z(i)} \lambda_i \,, \tag{16}$$

for all $i \in [1, 2^n - 1]$, where $x(i) + y(i) + z(i) \leqslant n$ is the number of non-identity terms in the i-th Pauli string. Noting that $x(i) + y(i) + z(i) \geqslant 1$ $\forall i$ and using Eq. (6), we obtain the desired statement. Equation (15) follows simply from explicit calculation of the vector norms. □

Supplementary Lemma 3. *(Action of noise + unitaries) Consider a channel*

$$\mathcal{W}^k = \mathcal{U}_k \circ \mathcal{N} \circ \cdots \circ \mathcal{N} \circ \mathcal{U}_2 \circ \mathcal{N} \circ \mathcal{U}_1 \circ \mathcal{N} \circ \mathcal{U}_0 \tag{17}$$

that consists of k *noise channels* $\mathcal{N}$ *of the form* (5) *interleaved with unitary channels* $\mathcal{U}_i$. *Consider the action of this channel on an operator* Λ *of the form* (9). *We have*

$$\left\|\mathcal{W}^k(\Lambda)\right\|_\infty \leqslant \lambda_0 + q^{2k} \left\|\boldsymbol{\lambda}\right\|_1 . \tag{18}$$

Proof. We have

$$\begin{aligned}
\left\|\mathcal{W}^k(\Lambda)\right\|_\infty &= \left\|\mathcal{W}^k\big(\lambda_0 \mathbb{1} + \sum_i \lambda_i \sigma_n^i\big)\right\|_\infty & (19)\\
&= \left\|\lambda_0 \mathbb{1} + \mathcal{W}^k\big(\sum_i \lambda_i \sigma_n^i\big)\right\|_\infty & (20)\\
&\leqslant \left\|\lambda_0 \mathbb{1}\right\|_\infty + \sum_i \left\|\mathcal{W}^k(\lambda_i \sigma_n^i)\right\|_\infty & (21)\\
&\leqslant \lambda_0 + q^{2k} \sum_i \left\|\lambda_i \sigma_n^i\right\|_\infty & (22)\\
&= \lambda_0 + q^{2k} \sum_i |\lambda_i| & (23)\\
&= \lambda_0 + q^{2k} \left\|\boldsymbol{\lambda}\right\|_1 , & (24)
\end{aligned}$$

where the first inequality is a series of triangle inequalities, the second inequality comes from repeated application of Supplementary Lemmas 1 and 2, and the third equality follows from the fact that the eigenvalues of a Pauli string are ± 1. □

Supplementary Lemma 4 (Wenzel/Audenaert [3], Theorem 3)**.** *(Schatten norms of commutators) Let* X *and* Y *be complex matrices. Let* $\|\cdot\|_p$, $\|\cdot\|_q$, $\|\cdot\|_r$, *denote the respective Schatten* p-, q-, r-*norms. The inequality*

$$\left\|[X, Y]\right\|_p \leqslant C_{p,q,r} \left\|X\right\|_q \left\|Y\right\|_r \tag{25}$$

holds for all (p, q, r) *satisfying* $\frac{1}{p} \leqslant \frac{1}{q} + \frac{1}{r}$, *excluding the octant* $p > 2$, $q < 2$, $r < 2$, *and where*

$$C_{p,q,r} = \max\left\{2^{1/p},\, 2^{1-1/q},\, 2^{1-1/r},\, 2^{1+1/p-1/q-1/r}\right\}. \tag{26}$$

The proof follows from an extensive investigation using complex interpolation theory, and can be found in Ref. [3].

Supplementary Lemma 5 (Müller-Hermes/França/Wolf [4], Theorem 6.1)**.** *(Evolution of relative entropy) Consider a channel*

$$\mathcal{W} = \mathcal{U}_k \circ \mathcal{N} \circ \cdots \circ \mathcal{N} \circ \mathcal{U}_2 \circ \mathcal{N} \circ \mathcal{U}_1 \circ \mathcal{N} \tag{27}$$

that consists of k noise channels $\mathcal{N} = \mathcal{N}_1 \otimes \ldots \otimes \mathcal{N}_n$ where each $\mathcal{N}$ is a depolarizing noise channel with depolarizing probability p, interleaved with unitary channels $\mathcal{U}_i$. Denote the relative entropy as $D\left(\cdot \middle\| \cdot \right)$. We have

$$D\left(\mathcal{W}(\rho) \middle\| \frac{\mathbb{1}^{\otimes n}}{2^n}\right) \leqslant (1-p)^{2k} D\left(\rho \middle\| \frac{\mathbb{1}^{\otimes n}}{2^n}\right) \leqslant (1-p)^{2k} n. \tag{28}$$

Supplementary Lemma 6. *Consider a single instance of the noise channel $\mathcal{N} = \mathcal{N}_1 \otimes \ldots \otimes \mathcal{N}_n$ where each local noise channel $\{\mathcal{N}_j\}_{j=1}^n$ is a Pauli noise channel that satisfies (5). Then, we have*

$$D_2\left(\mathcal{N}(\rho) \middle\| \frac{\mathbb{1}^{\otimes n}}{2^n}\right) \leqslant q^2 D_2\left(\rho \middle\| \frac{\mathbb{1}^{\otimes n}}{2^n}\right) . \tag{29}$$

where $D_2\left(\cdot \middle\| \cdot \right)$ denotes the sandwiched 2-Rényi relative entropy.

Proof. We note that this comes as a consequence of Corollary 5.6 of Ref. [5]. Let us first restate the result for convenience: For some density operator σ and $p > 0$ consider the channel $\mathcal{A}_{p,\sigma}(\cdot) = p(\cdot) + (1-p)\sigma$. Suppose that some other channel $\mathcal{B}$ satisfies

$$\left\| \Gamma_{\mathcal{B}(\sigma)}^{-\frac{1}{2}} \circ \mathcal{B} \circ \mathcal{A}_{p,\sigma}^{-1} \circ \Gamma_{\sigma}^{\frac{1}{2}} \right\|_{2\to 2} \leqslant 1 \tag{30}$$

where $\mathcal{A}_{p,\sigma}^{-1}$ denotes the inverse map of $\mathcal{A}_{p,\sigma}$ and Γ_σ^p denotes the map $\Gamma_\sigma^p(\cdot) = \sigma^{\frac{p}{2}}(\cdot)\sigma^{\frac{p}{2}}$. Then, for all states ρ,

$$D_2\left(\mathcal{B}^{\otimes n}(\rho) \| \mathcal{B}^{\otimes n}\left(\sigma^{\otimes n}\right)\right) \leqslant \alpha(p,\sigma) D_2\left(\rho \| \sigma^{\otimes n}\right) \tag{31}$$

where $\alpha(p,\sigma) = \exp\left(\left(1 - \left\|\sigma^{-1}\right\|^{-1}\right) \frac{\log(p)}{\log(\|\sigma^{-1}\|)}\right)$. If one chooses $\mathcal{A}_{p,\sigma}$ to be the depolarizing channel $\mathcal{D}_{p_d}$ with depolarizing probability p_d, then (31) implies that if some qubit channel $\mathcal{B}$ satisfies

$$\left\|\mathcal{B} \circ \mathcal{D}_{p_d}^{-1}\right\|_{2\to 2} \leqslant 1 . \tag{32}$$

then for any n-qubit states ρ we have

$$D_2\left(\mathcal{B}^{\otimes n}(\rho) \middle\| \frac{\mathbb{1}^{\otimes n}}{2^n}\right) \leqslant (1-p_d) D_2\left(\rho \middle\| \frac{\mathbb{1}^{\otimes n}}{2^n}\right) , \tag{33}$$

where we have used the standard inequality $\ln(x+1) \geqslant \frac{x}{x+1}$.

Now suppose that $\mathcal{B}$ is the qubit Pauli noise channel $\mathcal{B}$ as defined in (5). We can explicitly write the condition (32) as

$$\sup_{X \neq 0} \frac{\|\mathcal{B} \circ \mathcal{D}_{p_d}^{-1}(X)\|_2}{\|X\|_2} \leqslant 1. \tag{34}$$

We note that the superoperator (Pauli transfer matrix) of the concatenated channel $\mathcal{B} \circ \mathcal{D}_{p_d}^{-1}$ is diagonal with diagonal entries $(1, \frac{q_x}{1-p_d}, \frac{q_y}{1-p_d}, \frac{q_z}{1-p_d})$. Consider an arbitrary complex matrix X decomposed in the Pauli basis as $X = a\mathbb{1} + \boldsymbol{b} \cdot \boldsymbol{\sigma}$, where $\boldsymbol{\sigma}$ is the vector of Pauli matrices and $\boldsymbol{b}$ is a vector of complex coefficients. Then one can verify

$$\|X\|_2 = \sqrt{2}\sqrt{|a|^2 + \textstyle\sum_i |b_i|^2} , \tag{35}$$

$$\|\mathcal{B} \circ \mathcal{D}_p^{-1}(X)\|_2 = \sqrt{2}\sqrt{|a|^2 + \textstyle\sum_i \left(\frac{q_i}{1-p_d}\right)^2 |b_i|^2} , \tag{36}$$

where the second equality is obtained by reading off the diagonal entries of the superoperator of $\mathcal{B} \circ \mathcal{D}_{p_d}^{-1}$. In order to satisfy condition (34), one can pick

$$1 - p_d = \max_{i \in \{X,Y,Z\}} |q_i| . \tag{37}$$

Thus, by denoting $q = \sqrt{\max_{i \in \{X,Y,Z\}} |q_i|}$ and inspecting (33) we obtain the result as required. □

Finally, for convenience we quote two standard results and once again point the reader to [2] for further details.

Supplementary Lemma 7. *(Tracial matrix Hölder's inequality [6])*

Consider two $d \times d$ matrices A and B. Then we have

$$\left|\operatorname{Tr} A^{\dagger} B\right| \leqslant \|A\|_r \, \|B\|_s , \tag{38}$$

for all $1 \leqslant r, s \leqslant \infty$ such that $\frac{1}{r} + \frac{1}{s} = 1$.

Supplementary Lemma 8. *(Pinsker's inequality [7])*

Consider two quantum states $\rho, \sigma \in \mathcal{S}(\mathcal{H})$. Then, the quantum relative entropy $D(\rho\|\sigma)$ is lower bounded as

$$D(\rho\|\sigma) \geqslant \frac{1}{2\ln 2}\|\rho - \sigma\|_1^2 . \tag{39}$$

Supplementary Note 2 - Proof of Theorem 1

Here we provide the proof for our main result of Theorem 1, which we now recall for convenience.

Theorem 1 (Upper bound on the partial derivative)**.** *Consider an L-layered ansatz as defined in Eq. (4). Let θ_{lm} denote the trainable parameter corresponding to the Hamiltonian H_{lm} in the unitary $U_l(\boldsymbol{\theta}_l)$ appearing in the ansatz. Suppose that local Pauli noise of the form in Eq. (5) with noise parameter q acts before and after each layer as in Supplementary Figure 1. Then the following bound holds for the partial derivative of the noisy cost function*

$$|\partial_m \widetilde{C}| \leqslant F(n), \tag{40}$$

where

$$F(n) = \sqrt{8\ln 2}\, N_O \left\|\boldsymbol{\omega}\right\|_\infty \left\|H_{lm}\right\|_\infty n^{1/2} q^{L+1} \tag{41}$$

and where $\boldsymbol{\omega}$ is defined in Eq. (1), with respective number of non-zero elements N_O.

Proof. We write the overall channel that the state undergoes before measurement as the concatenation of two channels:

$$\mathcal{N} \circ \mathcal{U}_L(\boldsymbol{\theta}_L) \circ \cdots \circ \mathcal{N} \circ \mathcal{U}_2(\boldsymbol{\theta}_2) \circ \mathcal{N} \circ \mathcal{U}_1(\boldsymbol{\theta}_1) \circ \mathcal{N}\,(\cdot) = \mathcal{W}_a \circ \mathcal{W}_b(\cdot)\,, \tag{42}$$

where

$$\mathcal{W}_b = \mathcal{U}_m^{-}(\boldsymbol{\theta}_l) \circ \mathcal{N} \circ \mathcal{U}_{l-1}(\boldsymbol{\theta}_{l-1}) \circ \cdots \circ \mathcal{N} \circ \mathcal{U}_1(\boldsymbol{\theta}_1) \circ \mathcal{N}, \tag{43}$$

$$\mathcal{W}_a = \mathcal{N} \circ \mathcal{U}_L(\boldsymbol{\theta}_L) \circ \cdots \circ \mathcal{U}_{l+1}(\boldsymbol{\theta}_{l+1}) \circ \mathcal{N} \circ \mathcal{U}_m^{+}(\boldsymbol{\theta}_l)\,. \tag{44}$$

Here the define the unitary channels $\mathcal{U}_m^{-}(\boldsymbol{\theta}_l)$ and $\mathcal{U}_m^{+}(\boldsymbol{\theta}_l)$ that respectively correspond to the following unitaries:

$$U_m^{-}(\boldsymbol{\theta}_l) = \prod_{s=1}^{m} e^{-i\theta_{ls}H_{ls}}\,, \qquad U_m^{+}(\boldsymbol{\theta}_l) = \prod_{s>m} e^{-i\theta_{ls}H_{ls}}\,, \tag{45}$$

such that $U_m^{+}(\boldsymbol{\theta}_l)U_m^{-}(\boldsymbol{\theta}_l) = U_l(\boldsymbol{\theta}_l)$. For simplicity of notation let us denote $\partial_{lm}\widetilde{C} = \partial_{\theta_{lm}}\widetilde{C}$. We have

$$\partial_{lm}\widetilde{C} = \operatorname{Tr}[O\,\partial_{lm}\rho_L]\,, \tag{46}$$

with

$$\partial_{lm}\,\rho_L = \partial_{lm}\left(\mathcal{W}_a \circ \mathcal{W}_b(\rho_0)\right) \tag{47}$$

$$= \mathcal{W}_a\left(\partial_{lm}\,\bar{\rho}_l\right)\,, \tag{48}$$

where we denote $\bar{\rho}_l = \mathcal{W}_b(\rho_0)$. Thus we can write the derivative of the noisy cost function as

$$\left|\partial_{lm}\widetilde{C}\right| = \left|\operatorname{Tr}\left[\mathcal{W}_a^{\dagger}(O)\,\partial_{lm}\,\bar{\rho}_l\right]\right| \tag{49}$$

$$\leqslant \left\|\mathcal{W}_a^{\dagger}(O)\right\|_\infty \left\|\left(\partial_{lm}\,\bar{\rho}_l\right)\right\|_1 \tag{50}$$

where $\mathcal{W}_a^\dagger$ is the adjoint map of $\mathcal{W}_a$, and Eq. (50) comes from application of Hölder's inequality. We now upper bound both terms individually.

The first term can be bounded as

$$\big\|\mathcal{W}_a^\dagger(O)\big\|_\infty \leqslant q^{2(L-l+1)}\big\|\boldsymbol{\omega}\big\|_1 \tag{51}$$
$$\leqslant q^{2(L-l+1)} N_O \big\|\boldsymbol{\omega}\big\|_\infty . \tag{52}$$

The first inequality comes from application of Supplementary Lemma 3, additionally noting that $\omega_0 = 0$ and that Pauli channels are self-adjoint maps. The second inequality follows by using the bound $|\omega_i| \leqslant \big\|\boldsymbol{\omega}\big\|_\infty$.

Second, let us upper bound on the 1-norm of $\partial_{lm}\,\bar{\rho}_l = \partial_{lm}\mathcal{W}_b(\rho_0)$. We have

$$\partial_{lm}\,\bar{\rho}_l = \text{-}iH_{lm}\bar{\rho}_l + i\bar{\rho}_l H_{lm} \tag{53}$$
$$= -i\big[H_{lm}\,,\,\bar{\rho}_l\big] . \tag{54}$$

This enables us to write

$$\big\|\partial_{lm}\,\bar{\rho}_l\big\|_1 = \Big\|\big[H_{lm}\,,\,\bar{\rho}_l\big]\Big\|_1 \tag{55}$$
$$= \Big\|\Big[H_{lm}\,,\,\frac{1}{2^n}\big(\mathbb{1}^{\otimes n} + \bar{\boldsymbol{a}}^{(l)}\cdot\boldsymbol{\sigma}_n\big)\Big]\Big\|_1 \tag{56}$$
$$= \Big\|\Big[H_{lm}\,,\,\frac{1}{2^n}\big(\bar{\boldsymbol{a}}^{(l)}\cdot\boldsymbol{\sigma}_n\big)\Big]\Big\|_1 \tag{57}$$
$$\leqslant 2\,\Big\|\frac{1}{2^n}\big(\bar{\boldsymbol{a}}^{(l)}\cdot\boldsymbol{\sigma}_n\big)\Big\|_1\,\big\|H_{lm}\big\|_\infty \tag{58}$$
$$\leqslant 2\sqrt{2\ln 2\;D\Big(\bar{\rho}_l\Big\|\frac{\mathbb{1}}{2^n}\Big)}\,\big\|H_{lm}\big\|_\infty \tag{59}$$
$$\leqslant 2\sqrt{2\ln 2\;D_2\Big(\bar{\rho}_l\Big\|\frac{\mathbb{1}}{2^n}\Big)}\,\big\|H_{lm}\big\|_\infty \tag{60}$$
$$\leqslant \sqrt{8\ln 2\cdot q^{2l} n}\,\big\|H_{lm}\big\|_\infty \tag{61}$$
$$= \sqrt{8\ln 2}\,\big\|H_{lm}\big\|_\infty\, n^{1/2} q^{l} . \tag{62}$$

In the second equality we use the Pauli decomposition (8). In the third equality we use the fact that H_{lm} and $\mathbb{1}^{\otimes n}$ commute. The first inequality is due to application of Supplementary Lemma 4. In the second inequality we use Pinsker's inequality. The third inequality comes from the monotonicity of the sandwiched 2-Rényi relative entropy. The fourth inequality follows from repeated application of Supplementary Lemma 6 along with the data-processing inequality, and an upper bound on the 2-Rényi relative entropy that is saturated for pure states. The final line is simply a rearrangement of terms.

Inserting (52) and (62) into Eq. (50), we finally obtain

$$\big|\partial_{lm}\widetilde{C}\big| \leqslant \sqrt{8\ln 2}\,N_O\big\|\boldsymbol{\omega}\big\|_\infty\big\|H_{lm}\big\|_\infty n^{1/2} q^{L+1} , \tag{63}$$

as required, where we have loosened the bound by using $q^{2(L-l+1)} < q^{L-l+1}$ for $0 \leqslant q < 1$. □

1. *Stronger bound for low noise levels under more restrictive Pauli noise model*

We note that via alternative proof techniques one may obtain a similar bound to Theorem 1 for a different class of local Pauli noise models, where the bound is stronger in the regime of low noise strength (i.e., large q) and relatively uniform local Pauli error probabilities (i.e., close to local depolarizing noise). The core idea is that certain qubit Pauli channels can be decomposed into a depolarizing channel with non-trivial depolarizing probability, followed by a different Pauli channel.

Consider a unital Pauli channel $\mathcal{P}_{p_x,p_y,p_z}$ whose action on qubit state ρ takes the form

$$\mathcal{P}_{p_x,p_y,p_z}(\rho) = p_I\rho + p_x X\rho X + p_y Y\rho Y + p_z Z\rho Z . \tag{64}$$

where (p_I, p_x, p_y, p_z) is a probability vector. It can then be checked (e.g. by using the superoperator formalism) that

$$\mathcal{P}_{p_x,p_y,p_z} = \mathcal{P}_{p'_x,p'_y,p'_z} \circ \mathcal{D}_p \,. \tag{65}$$

is a valid decomposition of $\mathcal{P}_{p_x,p_y,p_z}$, where $\mathcal{D}_p$ is a depolarizing channel with depolarizing probability $p = 4\min(p_I, p_x, p_y, p_z)$ and $\mathcal{P}_{p'_x,p'_y,p'_z}$ is a Pauli channel with $p_j = \frac{p_j - p_i}{1-4p_i}$ for all $j \in \{I, X, Y, Z\}$ where $p_i = \min(p_I, p_x, p_y, p_z)$. The decomposition (65) allows us to directly use Supplementary Lemma 5 along with the data-processing inequality to modify Eqs. (59)-(61) to obtain the result

$$\left|\partial_{lm}\widetilde{C}\right| \leqslant \sqrt{8\ln 2}\, N_O \left\|\boldsymbol{\omega}\right\|_\infty \left\|H_{lm}\right\|_\infty n^{1/2}\hat{q}^{L+1} \,, \tag{66}$$

where $\hat{q} = 1 - 4\min(p_I, p_x, p_y, p_z)$. We note that set of Pauli noise models for which $p > 0$ in (65) is a strict subset of those for which $q < 1$ in (5).

Supplementary Note 3 - Proof of Lemma 1

In this section, we provide a proof for Lemma 1. We note that this Lemma is derived by employing techniques similar to those used in deriving Theorem 1 in Section B.

Lemma 1 (Concentration of the cost function). *Consider an L layer ansatz of the form in Eq. (4). Suppose that local Pauli noise of the form of Eq. (5) with noise strength q acts before and after each layer as in Supplementary Figure 1. Then, for a cost function $\widetilde{C}$ of the form in Eq. (3), the following bound holds*

$$\left|\widetilde{C} - \frac{1}{2^n}\mathrm{Tr}[O]\right| \leqslant G(n) \left\|\rho - \frac{\mathbb{1}}{2^n}\right\|_1 , \tag{67}$$

where

$$G(n) = N_O \|\boldsymbol{\omega}\|_\infty \, q^{2L+2} \,. \tag{68}$$

Here $\|\cdot\|_\infty$ is the infinity norm, $\boldsymbol{\omega}$ is defined in Eq. (1) and $N_O = |\boldsymbol{\omega}|$ is the number of non-zero elements in the Pauli decomposition of O.

Proof. We denote the overall channel that the state undergoes before measurement as $\mathcal{W}$. We can write

$$\widetilde{C} = \mathrm{Tr}[O\,\mathcal{W}(\rho)] \tag{69}$$

$$= \mathrm{Tr}[\mathcal{W}^\dagger(O)\,\rho] \tag{70}$$

$$= \frac{1}{2^n}\Big(\mathrm{Tr}\big[\mathcal{W}^\dagger(O)\big] + \mathrm{Tr}\big[\mathcal{W}^\dagger(O)\,\boldsymbol{a}^{(0)}\cdot\boldsymbol{\sigma}_n\big]\Big) \,, \tag{71}$$

where $\mathcal{W}^\dagger$ is the adjoint map to $\mathcal{W}$, and in the final line we used the Pauli decomposition (8). Note as we are dealing with Pauli noise and unitary operations, $\mathcal{W}^\dagger$ is a valid (trace preserving) channel composed of unitaries and Pauli noise. This enables us to write

$$\left|\widetilde{C} - \frac{1}{2^n}\mathrm{Tr}[O]\right| = \left|\mathrm{Tr}\Big[\mathcal{W}^\dagger(O)\,\big(\rho - \frac{\mathbb{1}}{2^n}\big)\Big]\right| \tag{72}$$

$$\leqslant \left\|\mathcal{W}^\dagger(O)\right\|_\infty \left\|\rho - \frac{\mathbb{1}}{2^n}\right\|_1 \tag{73}$$

$$\leqslant q^{2L+2}\left\|\boldsymbol{\omega}\right\|_1 \left\|\rho - \frac{\mathbb{1}}{2^n}\right\|_1 \tag{74}$$

$$\leqslant q^{2L+2} N_O \left\|\boldsymbol{\omega}\right\|_\infty \left\|\rho - \frac{\mathbb{1}}{2^n}\right\|_1 , \tag{75}$$

where the first inequality uses Hölder's inequality, the second inequality comes from application of Supplementary Lemma 3 and the second inequality follows by using the bound $|\omega_i| \leqslant \left\|\boldsymbol{\omega}\right\|_\infty$.

□

Supplementary Note 4 - Proof of Remark 1

We here present an extension to Theorem 1 to the case when several parameters in the ansatz $U(\boldsymbol{\theta})$ are correlated. Here, by correlated, we mean they are equal to each other [8]. Note that this is in contrast to the previously analyzed cases where we assumed that all parameters $\{\theta_{lm}\}_{lm}$ are independent. Specifically, Remark 1 provides an upper bound on the partial derivative of the cost function with respect to a parameter that is degenerate in $\boldsymbol{\theta}$.

Remark 1 (Degenerate parameters)**.** *Consider the ansatz defined in Eqs. (4). Suppose there is a subset G_{st} of the set $\{\theta_{lm}\}$ in this ansatz such that G_{st} consists of g parameters that are degenerate:*

$$G_{st} = \{\theta_{lm} \mid \theta_{lm} = \theta_{st}\} \tag{76}$$

Here, θ_{st} denotes the parameter in G_{st} for which $\|H_{lm}\|_\infty$ takes the largest value in the set. (θ_{st} can also be thought of as a reference parameter to which all other parameters are set equal in value.) Then the partial derivative of the noisy cost with respect to θ_{st} is bounded as

$$|\partial_{st}\widetilde{C}| \leqslant \sqrt{8\ln 2}\, g N_O \|H_{st}\|_\infty \|\boldsymbol{\omega}\|_\infty\, n^{1/2} q^{L+1}\,, \tag{77}$$

at all points in the cost landscape.

Proof. Using arguments similar to those in Section B, we get

$$|\partial_{st}\widetilde{C}| = \sum_{\theta_{hg}\in G_{st}} \left|\mathrm{Tr}[O\,\partial_{hg}\rho_L]\right| \tag{78}$$

$$\leqslant \sum_{\theta_{hg}\in G_{st}} \sqrt{8\ln 2} N_O \|H_{hg}\|_\infty \|\boldsymbol{\omega}\|_\infty\, n^{1/2} q^{L+1} \tag{79}$$

where the inequality was obtained from Eq. (66). Since there are g terms in the summation, we have

$$|\partial_{st}\widetilde{C}| \leqslant \sqrt{8\ln 2}\, g N_O \|H_{st}\|_\infty \|\boldsymbol{\omega}\|_\infty\, n^{1/2} q^{L+1}\,. \tag{80}$$

□

We note that the proof of Remark 1 can be trivially generalized to the case when the parameters in G_{st} are linear functions of the reference parameter.

Supplementary Note 5 - Proof of Remark 2

Remark 2 (Extensions to the noise model)**.** *We can extend our noise model to include additional non-local noise models and obtain the same scaling results. First, we may consider global (unital) Pauli noise $\mathcal{P}$ whose action on n-qubit Pauli string $\sigma_n \in \{\mathbb{1}, X, Y, Z\}^{\otimes n}$ can be written*

$$\mathcal{P}(\sigma_n) = q_{\sigma_n}\sigma_n \tag{81}$$

where $-1 \leqslant q_{\sigma_n} \leqslant 1$ for all σ_n, and $q_{\mathbb{1}^{\otimes n}} = 1$. Second, we can consider correlated coherent noise across multiple qubits of the form

$$\mathcal{V}(\rho) = V\rho V^\dagger \tag{82}$$

where $VV^\dagger = V^\dagger V = \mathbb{1}^{\otimes n}$. We can then consider a modification of our noisy cost function (3) as

$$\widetilde{C} \mapsto \widetilde{C}' = \mathrm{Tr}\Big[O\,\big(\mathcal{N}_L \circ \mathcal{U}_L(\boldsymbol{\theta}_L) \circ \mathcal{N}_{L-1} \circ \cdots \circ \mathcal{U}_2(\boldsymbol{\theta}_2) \circ \mathcal{N}_1 \circ \mathcal{U}_1(\boldsymbol{\theta}_1) \circ \mathcal{N}_0\big)(\rho_0)\Big] \tag{83}$$

with $\mathcal{N}_i = \mathcal{V}_i \circ \mathcal{P}_i \circ \mathcal{N}$ for all $i \in [0, L]$, where $\mathcal{P}_i$ and $\mathcal{V}_i$ are specific instances of global Pauli noise and correlated noise of the form of (81) and (82) respectively. Under such a modification, the statements of Lemma 1 and Theorem 1 still remain valid.

Proof. We can absorb the $\mathcal{V}_i$ channels into the parameterized unitaries and write

$$\widetilde{C}' = \mathrm{Tr}\Big[V_L^\dagger O V_L\ \left(\mathcal{N}_L' \circ \mathcal{Y}_L(\boldsymbol{\theta}_L) \circ \mathcal{N}_{L-1}' \circ \cdots \circ \mathcal{Y}_2(\boldsymbol{\theta}_2) \circ \mathcal{N}_1' \circ \mathcal{Y}_1(\boldsymbol{\theta}_1) \circ \mathcal{N}_0'\right)(\rho_0)\Big] \tag{84}$$

where $\mathcal{Y}_i(\boldsymbol{\theta}_i) = \mathcal{U}_i(\boldsymbol{\theta}_i) \circ V_{i-1}$ and $\mathcal{N}_i' = \mathcal{P}_i \circ \mathcal{N}$ for all $i \in [0, L]$. For any operator of the form (9), the effect of noise channel $\mathcal{N}_j$ is to map Pauli coefficients as

$$\lambda_i \xrightarrow{\mathcal{N}_j} q_X^{x(i)} q_Y^{y(i)} q_Z^{z(i)} \lambda_i\,, \tag{85}$$

for all $i \in [1, 2^n - 1]$, $j \in [0, L]$, where we can write $q = \max\{|q_X|, |q_Y|, |q_Z|\} < 1$. In addition, the results of Supplementary Lemma 3 are unchanged under the map $O \mapsto V_L^\dagger O V_L$. Thus, the above proofs proceed the same under such an extended noise model. □

Supplementary Note 6 - Proof of Corollaries 2 and 3

We first provide a proof of Corollary 2. We start by recalling that in the QAOA one sequentially alternates the action of two unitaries as

$$U(\boldsymbol{\gamma}, \boldsymbol{\beta}) = e^{-i\beta_p H_M} e^{-i\gamma_p H_P} \cdots e^{-i\beta_1 H_M} e^{-i\gamma_1 H_P}\,, \tag{86}$$

where H_P and H_M are the so-called problem and mixer Hamiltonian, respectively. We define N_P (N_M) the number of terms in the Pauli decompositions of H_P (H_M).

Corollary 2 (Example: QAOA)**.** *Consider the QAOA with $2p$ trainable parameters, as defined in Eq. (86). Suppose that the implementation of unitaries corresponding to the problem Hamiltonian H_P and the mixer Hamiltonian H_M require k_P- and k_M-depth circuits, respectively. If local Pauli noise of the form in Eq. (5) with noise parameter q acts before and after each layer of native gates, then we have*

$$|\partial_{\beta_l} \widetilde{C}| \leqslant \sqrt{8 \ln 2}\, g_{l,P} N_P \|H_P\|_\infty \|\boldsymbol{\omega}\|_\infty n^{1/2} q^{(k_P + k_M)p+1}\,, \tag{87}$$

$$|\partial_{\gamma_l} \widetilde{C}| \leqslant \sqrt{8 \ln 2}\, g_{l,M} N_P \|H_M\|_\infty \|\boldsymbol{\omega}\|_\infty\, n^{1/2} q^{(k_P + k_M)p+1}\,, \tag{88}$$

for any choice of parameters β_l, γ_l, and where $O = H_P$ in Eq. (2). Here $b_{l,P}$ and $b_{l,M}$ are respectively the number of native gates parameterized by β_l and γ_l according to the compilation.

Proof. We now treat each layer of native hardware gates as a unitary layer as in Supplementary Figure 1, which gives $L = (k_P + k_M)p$. In Eq. (80) we have $N_{st} = 1$, $\|\boldsymbol{\eta}_{\beta_l}\|_\infty \leqslant \|\boldsymbol{\eta}_P\|_\infty$, $\|\boldsymbol{\eta}_{\gamma_l}\|_\infty \leqslant \|\boldsymbol{\eta}_M\|_\infty$, assuming Trotterization. Then Corollary 2 follows by invoking Remark 1. □

Now let us provide a proof for Corollary 3. We recall that the UCC ansatz can be expressed as

$$U(\boldsymbol{\theta}) = \prod_{lm} U_{lm}(\theta_{lm}) = \prod_{lm} e^{i\theta_{lm} \sum_k \mu_{lm}^k \sigma_n^k}, \tag{89}$$

where $\mu_{lm}^k \in \{0, \pm 1\}$, and where θ_{lm} are the coupled cluster amplitudes. Moreover, we denote $\widehat{N}_{lm} = |\boldsymbol{\mu}_{lm}|$ as the number of non-zero elements in $\sum_k \mu_{lm}^k \sigma_n^k$.

Corollary 3 (Example: UCC)**.** *Let H denote a molecular Hamiltonian of a system of M_e electrons. Consider the UCC ansatz as defined in Eq. (89). If local Pauli noise of the form in Eq. (5) with noise parameter q acts before and after every $U_{lm}(\theta_{lm})$ in Eq. (89), then we have*

$$|\partial_{\theta_{lm}} \widetilde{C}| \leqslant \sqrt{8 \ln 2}\, \widehat{N}_{lm} N_H \|\boldsymbol{\omega}\|_\infty\, n^{1/2} q^{L+1}, \tag{90}$$

for any coupled cluster amplitude θ_{lm}, and where $O = H$ in Eq. (3).

Proof. Using the first-order Troterrization, the UCC ansatz can be represented as follows:

$$U(\boldsymbol{\theta}) = \prod_{lm} \prod_{k} e^{i\theta_{lm} \mu_{lm}^k \sigma_n^k}, \tag{91}$$

which is in the form of an ansatz that has correlated parameters. Then from Remark 1 it follows that

$$|\partial_{\theta_{lm}} \widetilde{C}| \leqslant \sqrt{8 \ln 2}\, \widehat{N}_{lm} N_H \|\boldsymbol{\omega}\|_\infty\, n^{1/2} q^{L+1}, \tag{92}$$

where we used the fact that in Eq. (80) $g = \widehat{N}_{lm}$, $N_{st} = 1$, and $\|\boldsymbol{\eta}_{st}\|_\infty = 1$ for the UCC ansatz as in Eq. (91). □

Supplementary Note 7 - Proof of Remark 3

Remark 3. *Suppose our noiseless cost function is instead*

$$C_{train} = \sum_i \mathrm{Tr}[O_i U(\boldsymbol{\theta})\rho_i U^\dagger(\boldsymbol{\theta})] \tag{93}$$

for a training set of data encoded in states $\{\rho_i\}$ and set of operators $\{O_i\}$ each of the form (2). Our result then generalizes to

$$\left|\partial_{lm}\widetilde{C}_{train}\right| \leqslant \sqrt{8\ln 2}\left(\sum_i N_{O_i}\left\|\boldsymbol{\omega}_i\right\|_\infty\right)\left\|H_{lm}\right\|_\infty n^{1/2}q^{L+1}\,. \tag{94}$$

Proof. We can denote each term in the sum (93) as $C_i = \mathrm{Tr}[O_i U(\boldsymbol{\theta})\rho_i U^\dagger(\boldsymbol{\theta})]$ such that

$$C_{\text{train}} = \sum_i C_i, \quad \widetilde{C}_{\text{train}} = \sum_i \widetilde{C}_i\,. \tag{95}$$

Then, we can simply write

$$\left|\partial_{lm}\widetilde{C}_{\text{train}}\right| = \left|\sum_i \partial_{lm}\widetilde{C}_i\right| \tag{96}$$

$$\leqslant \sum_i \left|\partial_{lm}\widetilde{C}_i\right|, \tag{97}$$

where each $\left|\partial_{lm}\widetilde{C}_i\right|$ can be bounded by Theorem 1 giving the result as required. □

Supplementary Note 8 - Proof of Proposition 1

In this Supplementary Note we provide a proof of Proposition 1, which we now recall for convenience.

Proposition 1 (Measurement noise)**.** *Consider the expansion of the observable O as a sum of Pauli strings, as in Eq. (2). Let w denote the minimum weight of these strings, where the weight is defined as the number of non-identity elements for a given string. In addition to the noise process considered in Supplementary Figure 1, suppose there is also measurement noise consisting of a tensor product of local bit-flip channels with bit-flip probability $(1-q_M)/2$. Then we have*

$$\left|\widetilde{C} - \frac{1}{2^n}\mathrm{Tr}\,O\right| \leqslant q_M^w\, G(n)\left\|\rho - \frac{\mathbb{1}}{2^n}\right\|_1 \tag{98}$$

and

$$|\partial_{lm}\widetilde{C}| \leqslant q_M^w F(n), \tag{99}$$

where $G(n)$ and $F(n)$ are defined in Lemma 1 and Theorem 1, respectively.

Proof. We prove in detail the proposition about the gradient of the cost function. The proposition about the cost function is derived in an analogous manner.

As a model of measurement noise we consider a classical bit-flip channel applied to every qubit, such that the standard POVM elements get replaced by:

$$P_0 = |0\rangle\langle 0| \to \widetilde{P}_0 = p_{00}|0\rangle\langle 0| + p_{01}|1\rangle\langle 1| \tag{100}$$

$$P_1 = |1\rangle\langle 1| \to \widetilde{P}_1 = p_{10}|0\rangle\langle 0| + p_{11}|1\rangle\langle 1|, \tag{101}$$

where $p_{00}+p_{01} = 1$ and $p_{10}+p_{11} = 1$. Furthermore, we take this channel to be unital, such that $\widetilde{P_0}+\widetilde{P_1} = (p_{00}+p_{10})P_0 + (p_{01}+p_{11})P_1 = P_0 + P_1$ giving $p_{00}+p_{10} = 1$ and $p_{01}+p_{11} = 1$. Thus, there is only one free

parameter q_M, and we set $p_{00} = p_{11} = \frac{1+q_M}{2}$, $p_{01} = p_{10} = \frac{1-q_M}{2}$. Note that without loss of generality we can assume $p_{00}, p_{11} > 1/2$, and hence $q_M \geqslant 0$. Overall:

$$P_0 = |0\rangle\langle 0| \to \widetilde{P}_0 = \frac{1+q_M}{2}|0\rangle\langle 0| + \frac{1-q_M}{2}|1\rangle\langle 1| \tag{102}$$

$$P_1 = |1\rangle\langle 1| \to \widetilde{P}_1 = \frac{1-q_M}{2}|0\rangle\langle 0| + \frac{1+q_M}{2}|1\rangle\langle 1| \,. \tag{103}$$

The equivalence between this classical channel and a quantum bit-flip channel is seen by writing $P_0 = \frac{\mathbb{1}+Z}{2}$ and $P_1 = \frac{\mathbb{1}-Z}{2}$, such that the bit-flip channel is equivalent to a transformation of the Pauli Z operator: $Z' = q_M Z$. This corresponds to the effect of a bit-flip channel $\mathcal{N}(\rho) = \frac{1+q_M}{2}\rho + \frac{1-q_M}{2}X\rho X$.

The reasoning so far only applies to measurements in the Z basis. However, in our model we do not consider a standard projective measurement, but the expectation value with respect to a general Hermitian operator. This assumes the capability of performing measurements in any basis. If we assume that the classical bit-flip acts independently of the basis we choose to measure in, then we see that the corresponding quantum channel must be a depolarizing channel such that

$$\mathcal{N}(\sigma) = q_M \sigma \,, \tag{104}$$

where σ is any single-qubit Pauli operator. An alternative realistic assumption that also leads to 104 is that the quantum computer can only measure in the computational basis, and so one implements measurements in general bases by applying an extra layer of (noisy) one-qubit rotations before measurement. We thus proceed to model measurement noise as a tensor product of such local depolarizing channels applied prior to measurement and denote the overall channel as $\mathcal{N}_M$. From Eq. (104) we have that

$$\mathcal{N}_M(O) = \sum_i \omega^i \mathcal{N}_M(\sigma_n^i) = \widetilde{\boldsymbol{\omega}} \cdot \boldsymbol{\sigma}_n \,, \tag{105}$$

where $\widetilde{\boldsymbol{\omega}}$ is a vector of elements $\widetilde{\omega}_i = q_M^{w(i)}\omega^i$, and where $w(i) = x(i) + y(i) + z(i)$ is the weight of the Pauli string. Here we recall that we have respectively defined $x(i)$, $y(i)$ and $z(i)$ as the number of Pauli operators X, Y, and Z in the i-th Pauli string. Let us now write the noisy cost function partial derivative as:

$$\partial_{lm}\widetilde{C} = \mathrm{Tr}\left[\mathcal{N}_M(O)\partial_{lm}\rho_L\right] \,. \tag{106}$$

Proceeding in the same way as in the proof of Theorem 1, we write

$$|\partial_{lm}\widetilde{C}| \leqslant \left\|\mathcal{W}_a^\dagger \circ \mathcal{N}_M(O)\right\|_\infty \left\|\partial_{lm}\,\bar{\rho}_l\right\|_1 \,. \tag{107}$$

The first term can be bounded as

$$\begin{aligned}
\left\|\mathcal{W}_a^\dagger \circ \mathcal{N}_M(O)\right\|_\infty &= \left\|\mathcal{W}_a^\dagger\left(\widetilde{\boldsymbol{\omega}} \cdot \boldsymbol{\sigma}_n\right)\right\|_\infty & (108)\\
&\leqslant \sum_i \left\|\mathcal{W}_a^\dagger\left(\widetilde{\omega}_i \sigma_n^i\right)\right\|_\infty & (109)\\
&= \sum_i q_M^{w(i)} \left\|\mathcal{W}_a^\dagger\left(\omega_i \sigma_n^i\right)\right\|_\infty & (110)\\
&\leqslant q_M^w \sum_i \left\|\mathcal{W}_a^\dagger\left(\omega_i \sigma_n^i\right)\right\|_\infty & (111)\\
&\leqslant q_M^w q^{L-l+1} N_O \left\|\boldsymbol{\omega}\right\|_\infty \,, & (112)
\end{aligned}$$

where the first inequality is due to the triangle inequality, the second equality comes from recalling the definition of $\widetilde{\boldsymbol{\omega}}$, the second inequality comes by using the definition of the minimum weight w, and the final equality follows by repeating the steps in the proof of Supplementary Lemma 3. We see this result is identical to that in Theorem 1, aside from an extra factor q_M^w.

The second term in (107) is bounded in the proof of Theorem 1 as

$$\left\|\partial_{lm}\,\bar{\rho}_l\right\|_1 \leqslant \sqrt{8\ln 2}\left\|H_{lm}\right\|_\infty n^{1/2} q^l \,. \tag{113}$$

Putting the two parts together we obtain

$$\left|\partial_{lm}\widetilde{C}\right| \leqslant \sqrt{8\ln 2}\, N_O \left\|\boldsymbol{\omega}\right\|_\infty \left\|H_{lm}\right\|_\infty n^{1/2}\, q_M^w\, q^{L+1} \tag{114}$$

$$= q_M^w F(n)\,, \tag{115}$$

as required.

Now let us prove the complimentary result for the cost function magnitude. Following the proof of Lemma 1 we write

$$\left|\widetilde{C} - \frac{1}{2^n}\mathrm{Tr}[O]\right| = \left|\mathrm{Tr}\left[\mathcal{W}^\dagger \circ \mathcal{N}_M(O)\left(\rho - \frac{\mathbb{1}}{2^n}\right)\right]\right| \tag{116}$$

$$\leqslant \left\|\mathcal{W}^\dagger \circ \mathcal{N}_M(O)\right\|_\infty \left\|\rho - \frac{\mathbb{1}}{2^n}\right\|_1 \tag{117}$$

$$\leqslant q_M^w\, q^{L+1}\left\|\boldsymbol{\omega}\right\|_1 \left\|\rho - \frac{\mathbb{1}}{2^n}\right\|_1 \tag{118}$$

$$\leqslant q_M^w\, q^{L+1} N_O \left\|\boldsymbol{\omega}\right\|_\infty \left\|\rho - \frac{\mathbb{1}}{2^n}\right\|_1 . \tag{119}$$

Thus we can write

$$\left|\widetilde{C} - \frac{1}{2^n}\mathrm{Tr}[O]\right| \leqslant q_M^w\, G(n)\left\|\rho - \frac{\mathbb{1}}{2^n}\right\|_1 , \tag{120}$$

where $G(n) = q^{L+1} N_O \left\|\boldsymbol{\omega}\right\|_\infty$. Hence observables with $w \in \Omega(n)$ will suffer from an exponential decay in n of the cost function and its gradient. □

[1] M. A. Nielsen and I. L. Chuang, *Quantum Computation and Quantum Information, Cambridge University Press* (2010).
[2] M. M. Wilde, *Quantum Information Theory*, 2nd ed. (Cambridge University Press, 2017).
[3] David Wenzel and Koenraad MR Audenaert, "Impressions of convexity: an illustration for commutator bounds," Linear algebra and its applications **433**, 1726–1759 (2010).
[4] Alexander Müller-Hermes, Daniel Stilck França, and Michael M Wolf, "Relative entropy convergence for depolarizing channels," Journal of Mathematical Physics **57**, 022202 (2016).
[5] Christoph Hirche, Cambyse Rouzé, and Daniel Stilck França, "On contraction coefficients, partial orders and approximation of capacities for quantum channels," arXiv preprint arXiv:2011.05949 (2020).
[6] Bernhard Baumgartner, "An inequality for the trace of matrix products, using absolute values," arXiv preprint arXiv:1106.6189 (2011).
[7] Masanori Ohya and Dénes Petz, *Quantum entropy and its use* (Springer Science & Business Media, 2004).
[8] Tyler Volkoff and Patrick J Coles, "Large gradients via correlation in random parameterized quantum circuits," Quantum Science and Technology (2021).
